# Large-Scale Survey Findings Inform Patients’ Experiences in Using Secure Messaging to Engage in Patient-Provider Communication and Self-Care Management: A Quantitative Assessment

**DOI:** 10.2196/jmir.5152

**Published:** 2015-12-21

**Authors:** Jolie N Haun, Nitin R Patel, Jason D Lind, Nicole Antinori

**Affiliations:** ^1^ Veterans Health Administration HSR&D Center of Innovation on Disability and Rehabilitation Research Tampa, FL United States; ^2^ Department of Community & Family Health College of Public Health University of South Florida Tampa, FL United States

**Keywords:** cross-sectional survey, email, Internet communication tools, veterans

## Abstract

**Background:**

Secure email messaging is part of a national transformation initiative in the United States to promote new models of care that support enhanced patient-provider communication. To date, only a limited number of large-scale studies have evaluated users’ experiences in using secure email messaging.

**Objective:**

To quantitatively assess veteran patients’ experiences in using secure email messaging in a large patient sample.

**Methods:**

A cross-sectional mail-delivered paper-and-pencil survey study was conducted with a sample of respondents identified as registered for the Veteran Health Administrations’ Web-based patient portal (My HealtheVet) and opted to use secure messaging. The survey collected demographic data, assessed computer and health literacy, and secure messaging use. Analyses conducted on survey data include frequencies and proportions, chi-square tests, and one-way analysis of variance.

**Results:**

The majority of respondents (N=819) reported using secure messaging 6 months or longer (n=499, 60.9%). They reported secure messaging to be helpful for completing medication refills (n=546, 66.7%), managing appointments (n=343, 41.9%), looking up test results (n=350, 42.7%), and asking health-related questions (n=340, 41.5%). Notably, some respondents reported using secure messaging to address sensitive health topics (n=67, 8.2%). Survey responses indicated that younger age (*P*=.039) and higher levels of education (*P*=.025) and income (*P*=.003) were associated with more frequent use of secure messaging. Females were more likely to report using secure messaging more often, compared with their male counterparts (*P*=.098). Minorities were more likely to report using secure messaging more often, at least once a month, compared with nonminorities (*P*=.086). Individuals with higher levels of health literacy reported more frequent use of secure messaging (*P*=.007), greater satisfaction (*P*=.002), and indicated that secure messaging is a useful (*P*=.002) and easy-to-use (*P*≤.001) communication tool, compared with individuals with lower reported health literacy. Many respondents (n=328, 40.0%) reported that they would like to receive education and/or felt other veterans would benefit from education on how to access and use the electronic patient portal and secure messaging (n=652, 79.6%).

**Conclusions:**

Survey findings validated qualitative findings found in previous research, such that veterans perceive secure email messaging as a useful tool for communicating with health care teams. To maximize sustained utilization of secure email messaging, marketing, education, skill building, and system modifications are needed. These findings can inform ongoing efforts to promote the sustained use of this electronic tool to support for patient-provider communication.

## Introduction

Patient-provider communication is central in delivering high quality of care and promoting positive patient outcomes [1]. Electronic asynchronous secure email messaging within Web-based patient portals is gaining popularity as a viable efficient form of patient-provider communication [2-4]. Secure email messaging is a priority in the United States, as part of a national transformation initiative to create new models of care to support patient-provider communication and promote self-care management within the context of the patient-centered medical home [5].

Electronic communication, such as secure messaging, has been shown to be effective in supporting self-care management, patient engagement, and efficient use of health services [2-4,6-8]. A systematic review of literature suggested that obtained data moderately support the use of secure messaging, to improve glucose outcomes and increase patient satisfaction, and that secure messaging as part of an electronic patient portal is more effective than secure messaging alone [9]. Although not as strong, some reports also suggest that there is some evidence that adding a Web-based pharmacist to secure messaging improves blood pressure outcomes in patients with hypertension, and that secure messaging within an electronic portal improves ulcerative colitis symptoms and adherence to colorectal cancer screenings or heart failure management [9]. Although some studies have seen a positive effect on utilization, it should be noted that the systematic review did find evidence that secure messaging may positively or negatively affect efficiency or utilization [9].

Recognizing that the implementation of secure messaging is widespread and is quickly becoming a common practice as part of services provided by integrated electronic patient portal services, efforts to understand patients’ experiences and needs when using secure messaging tools are warranted. This approach is imperative to supporting patients’ sustained use of electronic communication mechanisms, such as secure messaging.

Consistent with this consumer-centric approach, our previous qualitative research, consistent with other published studies, indicates patients’ value secure messaging as an efficient means of communication with their providers [10]. Benefits reported in a previous qualitative study included 24-hour access, avoiding phone calls and travel to health care facilities, and in general, saving time [10]. Our previous research provided insights into patients’ experiences, but larger quantitative data studies are needed to understand veterans’ experiences in using secure messaging and determine convergence with previous qualitative findings.

This paper presents findings of a cross-sectional survey study with a large sample of veterans who opted-in to use secure messaging to assess their reported experiences in using secure messaging and to evaluate factors that predict use and perceptions associated with using secure messaging. Findings from this survey research will inform efforts to quantify (1) veterans’ reported use of the secure messaging tool, (2) their reasons for using secure messaging, and (3) factors that influence their use of secure messaging. Gathering these data in a large representative sample can inform efforts to develop education and marketing content for potential users, identify points of intervention to support sustained secure messaging use, and continue the accumulation of reported evidence on the use of electronic forms of patient-provider communication.

## Methods

### Study Design Overview

This is a cross-sectional study. A paper-and-pencil survey via mail was sent to veterans who had registered for the Veteran Health Administrations’ Web-based patient portal (My HealtheVet) and opted to use secure messaging. The survey collected demographic data, assessed computer and health literacy, and secure messaging use.

### Setting and Participants

The two-site study was conducted at 2 large Department of Veterans Affairs (VA) Medical Centers (VAMCs): the James A. Haley Veterans' Hospital (Tampa, FL, USA) and the Veterans Affairs Boston Healthcare System (Boston, MA, USA). We used administrative data to identify veterans at both VAMCs who had registered for My HealtheVet, completed the in-person process of authenticating their identity, and accessed the system to “opt-in” to use secure messaging. We then used randomization to create contact lists of 2100 potential participants. Of the 2100, 2073 (1022 in Boston; 1051 in Tampa) had complete information to mail a survey for completion. Veterans received US $10 for completing the survey. This study was approved and regulated by the VA Central Institutional Review Board.

### Data Collection Instruments

The survey collected demographic data, assessed health literacy and eHealth literacy, and secure messaging use and perceptions. The majority of items were developed based on qualitative findings from a previous qualitative study conducted by the research team. Validated measures included the BRIEF Health Literacy Screening Tool [11,12], Computer-Email-Web (CEW) Fluency Scale [13], and The eHealth Literacy Scale (eHEALS) [14].

#### BRIEF Health Literacy Screening Tool

This is a 4-item screening tool to assess health literacy skills with 5-point Likert-type scale response options [11,12]. Score range was 4-20; score levels were as follows: 4-12=inadequate, 13-16=marginal, and 17-20=adequate. The correlation results of Rapid Estimate of Adult Literacy in Medicine (REALM) and Test of Functional Health Literacy in Adults-Short Form (STOFHLA) were as follows: *r*=.40, *P*<.01 for the BRIEF/REALM; *r*=.42, *P*<.01 for the BRIEF/STOFHLA; and *r*=.61, *P*<.01 for the REALM/STOFHLA. A principal component analysis suggested that the BRIEF measures one distinct construct “health literacy” (eigenvalue = 2.388), which accounted for 60% of score variance.

#### Computer-Email-Web Fluency Scale

The CEW Fluency Scale is an 18-item measure of common computer skills, with 5-point Likert-type scale response options (eg, not at all; very well) with a score range of 18-90. Cronbach alphas were established for subscales including computer fluency (alpha=.72), email fluency (alpha=.75), Web navigation (alpha=.64), and Web editing (alpha=.79) [13].

#### eHealth Literacy Scale

The eHEALS is a 10-item measure of eHealth literacy developed to measure consumers’ knowledge, comfort, and perceived skills at finding, evaluating, and applying electronic health information to health problems. eHEALS items have 5-point Likert-type scale response options, with a possible score range of 10-50. Previous validation results suggested internal consistency reliability to be .88, and test-retest reliability (*r*) from baseline to 6-month follow-up to be .68-.40. Principal components analysis produced a single factor (56% of variance) [14].

### Data Analysis

All survey data in this study were stored on a secure VA network. Analyses were managed using the statistical software suite SPSS (SPSS Inc, Chicago, IL, USA). Frequencies and proportions were computed for categorical variables and mean and standard deviations were computed for continuous variables to describe sample characteristics and provide a descriptive overview of survey findings. Chi-square tests were conducted to assess association between categorical variables and one-way analysis of variance was conducted to assess significant differences in means for continuous variables.

## Results

### Participants

Of the 2073 surveys mailed (1022 in Boston; 1051 in Tampa), 819 respondents provided completed survey data for analysis.

### Survey Findings

The majority of participants were older, white, non-Hispanic/non-Latino males, with an average age of 62 years (data not shown). Most participants had at least a high-school education and more than half (n=434, 53.0%) had an annual income of US $35,001 or more. Demographic data are presented in Table 1.

Most survey respondents reported everyday use of computer (n=662, 81.0%) and Internet (n=653, 79.7%). The majority of respondents reported using My HealtheVet a “few times a month or less” (n=629, 76.8%); and using secure messaging 6 months or longer (n=499, 60.9%). Most participants (486, 59.3%) reported using secure messaging at least once a year, and 131 (16.0%) reported using it at least once a month. Tables 2-5 present data on patients’ computer, Internet, My HealtheVet, and secure messaging use.

Pearson chi-square tests on demographic variables and secure messaging use indicate that younger age (*P*=.039) and higher levels of education (*P*=.025) and income (*P*=.003) are associated with more frequent use. Women were more likely to report using secure messaging more often (*P*=.098), but this only had a marginal significance. Minorities were more likely to report using secure messaging more often, at least once a month (*P*=.086). These findings and other demographic factors not significantly associated with secure messaging use are presented in Table 6.

Overall, respondents’ views on secure messaging are as follows: a good communication tool (n=619, 75.6%); saves time (n=590, 72.0%); and easy to use (n=544, 66.4%). Although 689 (84.1%) respondents reported intention to use secure messaging in the future, 342 (41.8%) reported that secure messaging could be improved to make it a more useful tool. Many respondents (n=328, 40.0%) reported that they would like to receive education and/or support on how to use My HealtheVet and secure messaging to manage their health care. A vast majority of respondents felt that other veterans would benefit from education on how to access and use My HealtheVet and secure messaging (n=652, 79.6%). The vast majority of respondents reported secure messaging as being a safe and secure form of communication (n=585, 71.4%). Patient-reported experiences in using secure messaging are illustrated in Table 7.

Respondents reported that secure messaging is useful for completing medication refills (n=546, 66.7%), medication questions (n=313, 38.2%), managing appointments (n=343, 41.9%), test results (n=350, 42.7%), and health-related questions (n=340, 41.5%). Notably, a small percentage of respondents reported using secure messaging to address sensitive health topics (n=67, 8.2%). Reasons why patients find it helpful and reasons for its use are presented in Table 8.

Secure messaging usefulness and reasons for its use scores were significantly higher for veterans who reported more frequent use of computer (*P*<.002 and *P*<.012, respectively) and Internet (*P*<.001 and *P*<.001, respectively). Similarly, scores were significantly higher for veterans who reported using My HealtheVet at least once a week (*P*<.001 and *P*<.001, respectively) and using secure messaging at least once a month (*P*<.001 and *P*<.001, respectively). Those who reported using secure messaging for more than a year also had significantly higher scores than those reporting its use for shorter periods. Findings related to respondents’ perceptions of usefulness and reasons for use by technology-use factors are presented in Table 9.

The BRIEF scores indicate that the majority of the sample had adequate health literacy (n=566, 69.1%); 174 (21.2%) had marginal health literacy and 77 (9.4%) had inadequate health literacy. The mean eHEALS and CEW Fluency Scale scores were 38.2 (SD 7.1; range 10-50) and 77.5 (SD 16.3; range 18-90), respectively. Respondents reporting higher levels of health literacy reported more frequent use of computers and the Internet (*P*≤.001), more frequent use of secure messaging (*P*=.007), and greater satisfaction with secure messaging (*P*=.002); additionally, they were more likely to report that it was a useful communication tool (*P*=.002), easy to use (*P*≤.001), and it as a safe and secure form of communication (*P*=.019). Interestingly, there were no differences in health literacy level based on reported intention to use secure email messaging in the future (*P*=.545). Those with lower levels of health literacy were more likely to request education and/or support (*P*≤.001). Individuals with higher eHEALS and CEW scores were also more likely to report more frequent use of computer, Internet, and secure email messaging (*P*≤.001 and *P*≤.001); greater satisfaction with the tool (*P*≤.001 and *P*=.013); they were also more likely to report that the tool was easy to use (*P*≤.001 and *P*≤.001), saves time (*P*≤.001 and *P*=.031), and is a safe and secure form of communication (*P*≤.001 and *P*≤.001). Individuals with higher eHEALS scores were also more likely to report intention to use secure email messaging in the future (*P*≤.001) and that secure email messaging was a useful communication tool (*P*≤.001). Statistics presenting relationships between respondents’ eHEALS, CEW, and BRIEF scores and secure messaging use and satisfaction are presented in Table 10.

**Table 1:** Descriptive statistics on patient demographics (N=819).

Variable		n (%)
**Study site**		
	Boston	339 (41.4)
	Tampa	480 (58.6)
**Gender**		
	Male	711 (86.8)
	Female	107 (13.1)
	Missing	1 (0.1)
**Minority status**		
	Minority	93 (11.4)
	Nonminority	726 (88.6)
**Ethnicity**		
	Hispanic or Latino	45 (5.5)
	Not Hispanic or Latino	706 (86.2)
	Missing	68 (8.3)
**Education**		
	High school or less	126 (15.4)
	Some college/vocational school/associate degree	380 (46.4)
	Bachelor's degree	198 (24.2)
	Graduate degree	113 (13.8)
	Missing	2 (0.2)
**Income**		
	≤US $15,000 per year	101 (12.3)
	US $15,001-US $25,000 per year	129 (15.8)
	US $25,001-US $35,000 per year	138 (16.8)
	US $35,001-US $45,000 per year	132 (16.1)
	>US $45,000 per year	302 (36.9)
	Missing	17 (2.1)
**Marital status**		
	Not married	333 (40.7)
	Married	483 (59.0)
	Missing	3 (0.4)

**Table 2:** Patients’ computer, Internet, and My HealtheVet use (N=819).

Response options	Never n (%)	Few times a month or less n (%)	At least once a week n (%)	Everyday n (%)
How often do you use a computer?	15 (1.8)	31 (3.8)	109 (13.3)	662 (80.8)
How often do you use the Internet?	15 (1.8)	33 (4.0)	116 (14.2)	653 (79.7)
How often do you use the My HealtheVet website?	37 (4.5)	629 (76.8)	136 (16.6)	10 (1.2)

**Table 3:** Data collection on participants’ My HealtheVet use (N=819).

Response options	Yes n (%)	No n (%)	I do not know n (%)
Have you completed the in-person authentication to upgrade your My HealtheVet account to use tools such as the secure messaging feature and Blue Button?	656 (80.1)	68 (8.3)	89 (10.9)
Have you opted-in to use the secure messaging feature on My HealtheVet?	658 (80.3)	70 (8.5)	86 (10.5)
Have you used the secure messaging (VA's secure email) feature on My HealtheVet?	565 (69.0)	204 (24.9)	45 (5.5)

**Table 4:** Participants’ secure messaging usage in general (N=819).

Response options	<6 months n (%)	6 months to 1 year n (%)	> 1 year n (%)	Does not apply n (%)
How long have you been using secure messaging?	171 (20.9)	187 (22.8)	312 (38.1)	133 (16.2)

**Table 5:** Participants’ secure messaging usage in My HealtheVet (N=819).

Response options	Never n (%)	At least once a year n (%)	At least once a month n (%)	Does not apply n (%)
How often do you use secure messaging on the My HealtheVet website?	116 (14.2)	486 (59.3)	131 (16.0)	76 (9.3)

## Discussion

### Principal Results

Findings from this survey research provided data on (1) veterans’ reported use of the secure messaging tool; (2) veterans’ reported reasons for using secure messaging; and (3) factors that influence their use of secure messaging. Key findings from this cross-sectional survey suggest that in a random sample (N=819) of patients receiving care within the VA who opted in to use secure messaging, a majority reported using secure messaging at least once a year, with less than 15% reporting never using the communication tool. Our VA sample had a higher percentage of participants reporting use of secure messaging than previous survey studies outside the VA, in which about 10-37% of respondents reported using email to contact their physician [15-17]. However, our percentage is likely higher due to our sampling methods that identified veteran patients who opted in to use secure messaging. It is safe to assume that this percentage would decrease if the survey were completed by the general patient population.

Overall, respondents reported being satisfied with secure messaging, as it provides a safe and secure communication tool that is easy to use and saves time. These results are consistent with our previous research and other reports [4,10,16,18,19]. Respondents reported that secure messaging is useful for tasks such as completing medication refills, managing appointments, receiving test results, and addressing health-related questions. A key finding in this study is that a small percentage of respondents reported using secure messaging to address sensitive health topics. This suggests that secure messaging offers patients a confidential, secure, and safe space to bring up sensitive topics, such as erectile dysfunction and sexually transmitted diseases, and avoiding the stigma or embarrassment of discussing these topics in person.

Some research suggests that patient concerns about data security may prevent the uptake of electronic health records; however, a majority of our respondents felt that secure messaging is a safe and secure form of communication [4,10,11].

Our findings are consistent with a previously published research outside the VA, which found that older age was negatively associated with frequency of use to contact health care providers using email (ie, secure messaging) [16]. Consistent with previous findings, income was positively correlated with preferences to use email (ie, secure messaging) to communicate with health care providers [15]. Previous research has shown mixed findings about minority groups’ preferences for using email-type services to communicate with health care providers [15,16]. However, in our sample, minority status was consistent with findings that suggest a positive association between minority status and use of electronic email communication with health care providers [16]. Further research is needed to better understand minority preferences and reasons for using electronic communication with health care providers.

**Table 6:** Pearson chi-square test between demographic variables and secure messaging use (N=819).

Demographic variable		How often do you use secure messaging?			
		Never	At least once a year	At least once a month	*P-*value
Age, mean (SD)		64.2 (13.1)	62.0 (12.9)	60.0 (13.1)	.039^a^
**Study site, n (%)**					
	Boston	56 (6.8)	190 (23.2)	55 (6.7)	.190
	Tampa	60 (7.3)	296 (36.1)	76 (9.3)	
**Gender, n (%)**					
	Male	106 (12.9)	415 (50.1)	109 (13.3)	.098^b^
	Female	9 (1.1)	71 (8.7)	22 (2.7)	
**White/Caucasian, n (%)**					
	No	14 (1.7)	48 (5.9)	22 (2.7)	.086^b^
	Yes	102 (12.5)	438 (53.5)	109 (13.3)	
**Ethnicity, n (%)**					
	Hispanic or Latino	7 (0.9)	24 (2.9)	8 (1.0)	.722
	Not Hispanic or Latino	97 (11.8)	431 (52.6)	108 (13.2)	
**Education, n (%)**					
	High school or less	28 (3.4)	66 (8.1)	25 (3.1)	.025^a^
	Some college/vocational School/associate degree	53 (6.5)	217 (26.5)	66 (8.1)	
	Bachelor's degree	23 (2.8)	123 (15.0)	26 (3.2)	
	Graduate degree	11 (1.3)	79 (9.6)	14 (1.7)	
**Income, n (%)**					
	≤US $15,000 per year	17 (2.1)	49 (6.0)	28 (3.4)	.003^a^
	US $15,001-US $25,000 per year	16 (2.0)	78 (9.5)	21 (2.6)	
	US $25,001-US $35,000 per year	29 (3.5)	79 (9.6)	19 (2.3)	
	US $35,001-US $45,000 per year	13 (1.6)	81 (9.9)	25 (3.1)	
	>US $45,000 per year	36 (4.4)	190 (23.2)	36 (4.4)	
**Marital status, n (%)**					
	Not married	48 (5.9)	203 (24.8)	60 (7.3)	.715
	Married	67 (8.2)	281 (34.3)	71 (8.7)	

^a^Significant at the .05 level.

^b^Significant at the .10 level.

**Table 7:** Patients’ experience in using secure messaging (N=819).

Experience	Disagree n (%)	Neutral n (%)	Agree n (%)	Do not know n (%)
I am satisfied with the secure messaging feature on My HealtheVet.	43 (5.3)	84 (10.3)	572 (69.8)	107 (13.1)
I get responses to my secure messages in a timely fashion.	51 (6.2)	84 (10.3)	515 (62.9)	154 (18.8)
Secure messaging is a useful tool to communicate with health care providers.	23 (2.8)	48 (5.9)	619 (75.6)	117 (14.3)
Secure messaging is easy to use.	61 (7.4)	88 (10.7)	544 (66.4)	112 (13.7)
Secure messaging saves patients’ time (eg, avoiding phone calls, and clinical visits).	30 (3.7)	70 (8.5)	590 (72.0)	115 (14.0)
Secure messaging could be improved to make it more useful to veterans.	62 (7.6)	215 (26.3)	342 (41.8)	186 (22.7)
Secure messaging is a secure and safe form of communication with VA providers.	18 (2.2)	71 (8.7)	585 (71.4)	134 (16.4)
I intend to use secure messaging in the future.	15 (1.8)	44 (5.4)	689 (84.1)	61 (7.4)
I would like to receive education and/or support on how to best use My HealtheVet and secure messaging to manage my health care.	185 (22.6)	246 (30.0)	328 (40.0)	49 (6.0)
Veterans would benefit from education on how to access and use My HealtheVet and secure messaging.	12 (1.5)	77 (9.4)	652 (79.6)	66 (8.1)

**Table 8:** Reasons patients find secure messaging helpful and reasons for use (N=819).

Reasons	Find secure messaging useful n (%)	Reason for using secure messaging n (%)
Medication refills	546 (66.7)	475 (58.0)
Medication questions	313 (38.2)	305 (37.2)
To manage appointments (eg, schedule, cancel)	343 (41.9)	301 (36.8)
Test results	350 (42.7)	292 (35.7)
Requests for tests	168 (20.5)	136 (16.6)
Request consult with specialist (eg, referral)	220 (26.9)	203 (24.8)
Health-related questions	340 (41.5)	301 (36.8)
Sensitive health topics (eg, sexually transmitted infections, mental health)	67 (8.2)	66 (8.1)
Can contact providers on my own time	425 (51.9)	381 (46.5)
Saves time compared with other method of communication (eg, phone)	448 (54.7)	377 (46.0)

In our study, respondents reporting higher levels of health literacy (BRIEF) and eHealth literacy (eHEALS and CEW) reported more frequent use of secure email messaging and greater satisfaction with secure messaging; besides, they were more likely to report that it was a safe, secure, and a useful communication tool. These findings are consistent with existing literature, suggesting that eHealth users tend to have higher levels of eHealth and health literacy [13,14,16,17,20].

Individuals with higher eHEALS were also more likely to report an intention to use secure email messaging in the future. Individuals with lower levels of health literacy were more likely to report a need for more education and/or support. Screening patients for their health literacy and eHealth literacy level may be an effective way to identify veterans with greater educational needs, and to allocate resources to support their use of tools such as secure messaging.

**Table 9:** Respondents’ perceptions of usefulness and reasons for use by technology-use factors are presented (N=819).

		Reasons for usefulness of secure messaging			Reasons for which messaging is used		
		n	Mean (SD)	*P*	n	Mean (SD)	*P*
**Frequency of computer use**				.002			.012
	Never	15	2.67 (1.72)		15	2.2 (1.66)	
	Few times a month or less	31	2.74 (2.68)		31	1.9 (2.39)	
	At least once a week	109	3.64 (2.75)		109	2.61 (2.40)	
	Everyday	662	4.17 (2.74)		662	3.08 (2.45)	
**Frequency of Internet use**				.001			.001
	Never	15	2.33 (1.72)		15	1.87 (1.69)	
	Few times a month or less	33	2.82 (2.92)		33	1.82 (2.37)	
	At least once a week	116	3.61 (2.67)		116	2.57 (2.32)	
	Everyday	653	4.19 (2.73)		653	3.1 (2.46)	
**Frequency of My HealtheVet use**				<.001			<.001
	Never	37	1.05 (2.4)		37	0.49 (1.37)	
	Few times a month or less	629	3.9 (2.63)		629	2.82 (2.32)	
	At least once a week	136	5.34 (2.51)		136	4.21 (2.45)	
	Everyday	10	4.3 (3.59)		10	3.7 (3.59)	
**Frequency of secure messaging use**				<.001			<.001
	Never	116	1.81 (2.47)		116	0.72 (1.51)	
	At least once a year	486	4.5 (2.35)		486	3.43 (2.13)	
	At least once a month	131	5.56 (2.36)		131	4.63 (2.34)	
**Length of secure messaging use**				<.001			<.001
	<6 months	171	3.82 (2.45)		171	2.75 (2.28)	
	6 months to 1 year	187	4.47 (2.4)		187	3.44 (2.27)	
	>1 year	312	4.97 (2.5)		312	3.84 (2.27)	

A vast majority (80%) of respondents felt that other veterans would benefit from education on how to access and use My HealtheVet and secure messaging. Furthermore, data suggest respondents’ perceptions of the usefulness of secure messaging are associated with frequency of use. These data warrant consideration for marketing secure messaging and providing education to intended users to ensure audiences understand the benefits and purposes for using this electronic communication tool. Finally, though the vast majority of participants were satisfied with the tool and reported intention to use secure messaging in the future, more than 40% reported that secure messaging tool could be improved to make it even more useful. This finding is timely and should be strongly considered as the VA continues efforts in redesigning and enhancing available electronic resources for their patients to support sustained use.

**Table 10:** Relationship between survey respondents’ eHEALS, CEW, and BRIEF scores and My HealtheVet and secure messaging use.

		eHealth literacy score			Computer-Email-Web Fluency score			BRIEF Health Literacy score		
		n	Mean (SD)	*P*	n	mean (SD)	*P*	n	mean (SD)	*P*
**Frequency of computer use**				<.001			<.001			<.001
	Never/few times per month or less	46	29.7 (7.5)		46	47.5 (24.1)		46	13.2 (4.6)	
	At least once a week	108	34.8 (7.3)		108	67.4 (17.1)		109	16.3 (3.1)	
	Everyday	661	39.4 (6.5)		661	81.2 (12.2)		662	17.8 (2.9)	
**Frequency of Internet use**				<.001			<.001			<.001
	Never/few times per month or less	48	29.3 (7.3)		48	47.9 (23.6)		48	13.4 (4.4)	
	At least once a week	115	34.6 (7)		115	67.1 (16.5)		116	16.2 (3.3)	
	Everyday	652	39.5 (6.4)		652	81.5 (12.1)		653	17.9 (2.8)	
**Frequency of My HealtheVet website use**				<.001			.023			.998
	Never/few times per month or less	664	37.8 (7.3)		664	77 (16.6)		664	17.4 (3.2)	
	At least once a week/everyday	146	40.6 (5.8)		146	80.4 (13.3)		146	17.4 (3.3)	
**Frequency of secure messaging use**				<.001			<.001			<.007
	Never	116	34.8 (8.3)		115	70.2 (20.2)		116	16.8 (3.3)	
	At least once a year	484	38.9 (6.5)		486	79.3 (14.6)		484	17.6 (3.1)	
	At least once a month	131	40 (6.1)		131	78.9 (14.5)		131	17 (3.5)	
**Satisfied with secure messaging tool**				<.001			.013			.002
	Disagree	43	36.1 (7.6)		43	73.1 (20.5)		43	16.5 (3.7)	
	Neutral	84	35.5 (7.1)		84	76.2 (15.8)		84	16.5 (3.7)	
	Agree	570	39.3 (6.4)		571	79.2 (14.5)		570	17.6 (3.1)	
**Secure messages receive response in a timely fashion**				<.001			.001			<.001
	Disagree	51	36.5 (7.5)		51	73.2 (16.4)		51	16.1 (3.4)	
	Neutral	84	35.6 (7.6)		84	75.9 (15.7)		84	16.7 (3.7)	
	Agree	513	39.7 (6.3)		515	80.1 (13.9)		513	17.8 (3.1)	
**Secure messaging is a useful communication tool**				<.001			.092			.002
	Disagree	23	34.7 (8.9)		23	76.2 (17.4)		23	16.4 (3.9)	
	Neutral	48	35.3 (5.9)		48	74.3 (15.8)		48	16.1 (4.2)	
	Agree	617	39.2 (6.6)		618	79 (15)		617	17.6 (3.1)	
**Secure messaging is easy to use**				<.001			<.001			<.001
	Disagree	61	34.7 (6.8)		61	70.9 (20.8)		61	15.9 (4.2)	
	Neutral	87	35.3 (6.7)		88	73.6 (16.2)		88	17.2 (2.9)	
	Agree	543	39.7 (6.3)		544	80.3 (13.6)		542	17.6 (3.1)	
**Secure messaging saves time**				<.001			.031			.084
	Disagree	30	35.2 (7.9)		30	75.4 (14.5)		30	16.4 (3.8)	
	Neutral	70	36 (6.8)		70	74.6 (17.8)		70	17 (3.2)	
	Agree	588	39.2 (6.6)		589	79.1 (14.7)		588	17.5 (3.2)	
**Secure messaging could be improved to make it more useful**				.001			.014			.031
	Disagree	62	41.4 (7)		62	83.5 (9.9)		62	18 (3.2)	
	Neutral	215	38.9 (6.5)		215	78.1 (15.9)		215	17.5 (3)	
	Agree	341	37.9 (7.1)		342	77.3 (16)		340	17 (3.4)	
**Secure messaging is a secure and safe form of communication**				<.001			.001			.019
	Disagree	18	37.4 (8.5)		18	77.6 (16.2)		18	16.3 (3.8)	
	Neutral	70	33.3 (8.3)		71	71.9 (18.6)		71	16.5 (3.8)	
	Agree	584	39.4 (6.2)		585	79.1 (14.8)		584	17.5 (3.2)	
**Intention to use secure messaging in the future**				<.001			.059			.545
	Disagree	15	34.1 (9.8)		15	73.9 (18.5)		15	17.4 (3.1)	
	Neutral	44	34.5 (9.1)		44	73.2 (19.9)		44	16.8 (4.2)	
	Agree	687	38.8 (6.6)		688	78.4 (15.3)		687	17.4 (3.2)	
**Education and/or support on how to best use My HealtheVet and secure messaging would be helpful**				<.001			<.001			<.001
	Disagree	184	41.2 (7)		183	83.4 (10.8)		185	18.3 (3)	
	Neutral	245	38.8 (6.9)		246	79.3 (14.7)		246	17.5 (3.2)	
	Agree	328	36.2 (6.9)		328	73.2 (18)		326	16.8 (3.2)	
**Veterans would benefit from education on how to access and use My HealtheVet and secure messaging**				.372			.027			.022
	Disagree	12	37.3 (11)		12	84.7 (9.2)		12	15.5 (4.8)	
	Neutral	77	39.3 (6.5)		77	81.2 (12.8)		77	18 (3.3)	
	Agree	651	38.1 (7.2)		651	76.9 (16.6)		651	17.2 (3.2)	

### Limitations

The limitations of this cross-sectional survey study should be considered when interpreting these data. First, the generalizability of the survey sample in our study is a strength and a limitation. Our data are representative of the veteran patient population who are registered and opted-in to use secure messaging; however, these data do not represent those veterans who did not opt in to use this communication tool nor represent the general population’s use of secure messaging systems outside of the VA. Furthermore, the response rate of our survey was less than 50%. Although consistent with response rates in similar user experience studies, caution should be exercised when generalizing these survey results to any veteran population. Second, our respondents were also more likely to be older white males, with higher levels of income and education. Although this is representative of the current veteran population, it is not representative of the diversification seen in younger active military and new veteran populations. Thus, it is best to exercise caution in generalizing our results to the entire veteran population; however, we can still draw useful conclusions from the survey data to understand veterans’ experiences and reasons for use of secure messaging to inform future research in evaluating and increasing the sustained meaningful use of secure messaging. Third, as with any cross-sectional study, this survey does not allow statements on the causality of secure messaging use, however, it does provide much needed descriptive data to understand veteran’s experiences in using secure messaging to manage their health. Finally, although this cross-sectional survey study provided important data on veterans’ experiences and use of secure messaging, we cannot comment on how clinicians and other VA health care team members are using secure messaging to reciprocate communication with their patients or their experiences in using this tool. These questions require further examination.

### Conclusions

Findings from this survey research provided data on veterans’ reported use of the secure messaging tool, their reasons for using secure messaging, and factors that influence their use of secure messaging. These large-scale survey findings validated previously published qualitative findings suggesting that veterans perceive secure messaging as a useful tool for communicating with health care teams. Secure messaging use, perceptions of ease of use, and satisfaction differ by gender, education, income, health, and eHealth literacy levels. These data contribute to the body of knowledge on the use of electronic forms of patient-provider communication such as secure messaging and can be used to inform efforts to develop education and marketing content for potential users, as well as identify points of intervention to support sustained secure messaging use.

## Abbreviations

CEW: Computer-Email-Web Fluency Scale
eHEALS: eHealth Literacy Scale
REALM: Rapid Estimate of Adult Literacy in Medicine
STOFHLA: Test of Functional Health Literacy in Adults-Short Form
VA: Department of Veterans Affairs
VAMC: VA Medical Centers
